# A programme to treat chronic hepatitis B in Kiribati: progress and challenges

**DOI:** 10.5365/wpsar.2019.10.4.003

**Published:** 2020-09-30

**Authors:** Alice U. Lee, Kathy Jackson, Rosemary Tekoaua, Caroline Lee, Margaret Sue Huntley, David C. Hilmers

**Affiliations:** a University of Sydney, Concord Repatriation General Hospital, Department of Gastroenterology and Liver Services, Concord, New South Wales 2139, Australia.; b Victorian Infectious Diseases Reference Laboratory, Royal Melbourne Hospital, The Peter Doherty Institute for Infection and Immunity, Victoria, Australia.; c Ministry of Health and Medical Services, Bikenibeu, Tarawa, Kiribati.; d University of New South Wales, Sydney, New South Wales, Australia.; e Hepatitis B Free, Sydney, New South Wales, Australia.; f Baylor College of Medicine, Departments of Internal Medicine, Pediatrics, Space Medicine and Global Initiatives, Houston, Texas, United States of America.

## Abstract

**Problem:**

Over 290 million people worldwide suffer from chronic hepatitis B (CHB), with the highest prevalence in the Pacific islands. Mortality attributable to this disease exceeds that from HIV, tuberculosis and malaria combined in this region.

**Context:**

CHB is a major health problem in the Pacific island nation of Kiribati. Medical care is complicated by vast expanses of ocean separating population centres in its constituent islands. Birth-dose hepatitis B immunization rates need improvement. High rates of obesity, metabolic syndrome, and co-infection with hepatitis B and hepatitis D in Kiribati make treatment less effective. Staff allocation, training and retention are difficult. Limited infrastructure creates challenges in training, communications, laboratory testing and record-keeping.

**Action:**

We have established a CHB treatment programme in Kiribati based on World Health Organization (WHO) guidelines and local needs. It includes direct patient care; laboratory, radiology and pharmacy support; public education; training; and data management. Thousands of individuals have been screened, and 845 hepatitis B-positive patients have had blood sent to Australia for molecular testing. Patient education pamphlets, medical training programmes and treatment protocols have been developed. Seventy-nine patients have started treatment. Regular onsite visits by technical experts are scheduled throughout the year.

**Lessons learnt and discussion:**

This is the first national CHB treatment programme established in the Pacific islands region. Unique challenges exist in Kiribati, as they do in each nation affected by CHB. Close engagement with local partners, knowledge of the barriers involved, flexibility, advocacy, and support from WHO and volunteer technical experts are key attributes of a successful treatment programme.

Chronic hepatitis B (CHB) is one of the leading causes of morbidity and mortality in the world. An estimated 2 billion people globally have been infected with hepatitis B sometime during their lives, and almost 300 million people suffer from CHB. (*1*) Nearly 900 000 chronically infected patients die each year, mainly due to liver failure, complications of cirrhosis such as variceal bleeding, and hepatocellular carcinoma. (*2*) A strategy to eliminate viral hepatitis as a public health threat by 2030 was adopted by the World Health Assembly in 2016. (*3*) Low- and middle-income countries (LMICs) face a particularly daunting task and are unlikely to reach this goal without significant effort, support and funding.

Highly effective antiviral treatment for CHB has been available for over 20 years, but it remains inaccessible in most resource-poor areas. Barriers include the high cost of drugs, the need for lifelong therapy in most patients (unlike hepatitis C therapy), the lack of infrastructure such as laboratory facilities, and a dearth of trained medical personnel. Furthermore, HIV, malaria and tuberculosis (TB) programmes compete for available resources, both global and domestic.

Pacific island nations have among the highest prevalence of hepatitis B infection in the world, in some places in excess of 20%. (*4*) The human suffering and economic costs associated with this disease are considerable and often underestimated. One of the first CHB treatment programmes in the Pacific islands was facilitated beginning in 2018 through a collaboration among the Kiribati Ministry of Health and Medical Services (MHMS), the World Health Organization (WHO), the Victorian Infectious Disease Reference Laboratory (VIDRL), and Hepatitis B Free (an Australian nongovernmental organization, known as HBF) and its partners, with the support of Australian Aid. We describe the progress made and the barriers encountered and addressed, and conclude that successful CHB treatment programmes in Pacific island countries, such as Kiribati, are challenging but achievable.

## Local context

Kiribati is a Pacific island nation of 116 000 people stretching across 3000 km of ocean approximately halfway between Australia and Hawaii. Distances between its islands make accessibility to services challenging. Half of the population lives on the island of South Tarawa. The lack of both arable land and dietary diversity result in high rates of diabetes, hypertension and obesity. (*5*)

In Kiribati, free health care is provided by MHMS. However, there is a shortage of health-care professionals, with fewer than 0.4 physicians per 1000 population, most of whom practise in South Tarawa. (*6*) Tungaru Central Hospital (TCH) on South Tarawa is the central referral facility. Health centres in the outer islands, staffed by nurses who receive additional training, offer primary care, midwifery services and medications. These services are supplemented by outreach clinics from TCH. Traditional medicine is also used, with practitioners providing local remedies and midwifery. (*7*)

Hospital laboratories performed over 10 000 hepatitis B surface antigen (HBsAg) tests per year between 2012 and 2014, with a seropositivity rate of 14–15%. (*7*) Nearly half of HBsAg-positive patients in Kiribati also have detectable hepatitis D virus (HDV) co-infection, which is problematic as co-infected patients are at higher risk for rapid progression to liver failure and have a poor response to antiviral therapy. (*8*)

## Action

In Kiribati, alarmingly high rates of CHB motivated the inclusion of antiviral treatment into a national hepatitis strategy. Based on an assessment by the WHO Regional Office for the Western Pacific, MHMS, with assistance from HBF and VIDRL, began developing a treatment programme.

HBF is composed of volunteer health professionals and non-medical personnel who have worked in the Pacific and Asia since 2013. It has addressed gaps in hepatitis B care through prevention, public education and advocacy initiatives; health-care worker training; and test-and-treat programmes in underserved communities. VIDRL is a public health and reference laboratory in Melbourne, Australia, that provides molecular diagnostic testing and local laboratory systems support. It holds the designation of a WHO Collaborating Centre for Viral Hepatitis and is the Regional Reference Laboratory for hepatitis B and D.

A memorandum of understanding to begin a treatment programme was signed by MHMS, TCH and HBF in 2017. Using experience gained from the development of CHB programmes in other countries, HBF developed a treatment protocol, and donations to provide medications were secured. Shortly thereafter, an import license was issued, and the antiviral medication tenofovir disoproxil fumarate (TDF) arrived in country. In January 2018, the first cohort of patients was examined and assessed for treatment eligibility, and staff were trained. In March 2018, the first patients began treatment. Within one year, 79 patients in Kiribati were receiving TDF, and hepatitis B and D viral load testing was performed on over 800 CHB-positive individuals.

Treatment protocols in Kiribati are based on WHO guidelines. (*9*) A culturally appropriate booklet that outlines the causes, complications, transmission and treatment of hepatitis B was developed jointly with health-care workers and is given to patients. All patients identified as HBsAg-positive are invited for further assessment at TCH. A history and physical examination are followed by laboratory testing, ultrasound evaluation and transient elastography. The latter is facilitated through equipment hand-carried by HBF volunteers during regular visits. Blood samples are batched for shipment to VIDRL for molecular diagnostics.

Patients with cirrhosis are prioritized for treatment, following identification by laboratory testing, a history and a physical exam (jaundice, variceal bleeding, encephalopathy), and transient elastography > 11.0 kPa, or ultrasound. Other priority groups include older patients with elevated alanine aminotransferase (ALT) and high viral load, patients with a strong family of liver cancer, and health-care workers.

Treatment candidates are counselled on their suitability for therapy versus monitoring. Those qualifying for treatment are asked to sign an informed-consent document and an agreement that states their intention to comply with the programme requirements. Generally, patients are given a 30-day supply of TDF at a time for the first several months of treatment. However, factors that affect adherence such as remote location, family hardship, age or physical disability may require dispensing several months’ worth of medication at once. Follow-up visits with laboratory and radiographic monitoring are scheduled according to WHO guidelines. (*9*)

The baseline characteristics of patients on treatment in Kiribati are shown in **Table 1**. Of note is male predominance (over two thirds), obesity (median body mass index of 31.2), elevated liver enzymes, high rates of co-infection with HDV (46.3%), and a preponderance of patients with cirrhosis, 56 (70.9%), by transient elastography.

**Table 1:** Characteristics of patients on HBV antiviral treatment in Kiribati, 2018–2019

Number of people on treatment	79
Age in years, median (range)	36.2 (22–58)
Sex, number (%)	54 (68.3%) Male 25 (31.7%) Female
BMI, mean (standard deviation)	31.2 (± 5.9)
Transient elastography (kPa), median (range)	15.2 (4.8–74.8)
APRI score,† median (range)	2.58 (0.18–33.1)
FIB4 score,‡ median (range)	3.22 (0.22–29.53)
AST (U/L), median (range)	61.5 (3–1934)
HBV viral load (IU/mL) in patients tested, median (range)	366 (15–3.25 × 10^8^)
Proportion HBsAg-positive with detectable HDV RNA (%)	46.3%

† APRI (AST to Platelet Ratio Index) Score = [(AST/ULN AST) × 100]/Platlets (109/L)]

‡ FIB4 (Fibrosis-4) Score = (Age x AST)/(Platelets (109/L) x (sqrt (ALT))

## Programme challenges

Initial successes have been tempered by several challenges, notably staff shortages. High health worker turnover disrupts programme continuity and necessitates frequent retraining. There is a paucity of medical staff, and most doctors are required to attend to multiple duties, which places them under great pressure at work. Regular visits by HBF volunteers are required to support the programme, but entry is difficult due to infrequent and expensive air service. As local medical staff also work on remote islands, attendance during training visits is not always possible. Limited Internet bandwidth has impacted training via teleconference.

Although over 800 patients have had viral load testing, many have yet to be evaluated in a clinic. Patient recall is difficult due to staff and clinic limitations, and many patients lack a mailing address. Patients living on the outer islands must journey by boat to attend clinics at TCH and may lack accommodations. Radio and social media are often used to disseminate clinic schedules and appointments due to lack of other forms of communication, contributing to inconsistent attendance and difficulty maintaining confidentiality.

Patient non-adherence with clinic visits is common. Local physicians note that patients will sometimes stop long-term medications due to family pressures, consultations with local traditional healers or lack of confidence in the local health system.

High rates of obesity complicate patient treatment. Fibrosis from metabolic syndrome and the hepatitis B virus (HBV) mono-infection or HBV/HDV co-infection constitute synergistic risks for the progression of liver disease. Dietary change is difficult given a dearth of arable land, low per capita income (*10*) and a lack of healthy food choices.

Recent availability of a hepatitis B cartridge for the GeneXpert (Cepheid, Sunnyvale, California, United States of America) machine has made routine access to viral load testing possible, but this has not been introduced in Kiribati. TCH has such technology for HIV and TB testing, but consumable costs, trained personnel, the need for safe disposal and allocated machine time to perform hepatitis B testing are problematic.

Medical documentation is challenging. Records for patients on TDF must be compiled from several different sources, including paper-based clinic records, laboratory results from Kiribati and VIDRL, and pharmacies. Overburdening of local staff and competing priorities delay data entry, making programme oversight challenging.

## Lessons learnt and interventions

Sensitivity to the local context has resulted in changes in programme strategies. A volunteer gastroenterologist and project manager from HBF have been designated as coordinators to help provide continuity in the programme. They make regular visits (two to three times a year) that have been extended to a week or more to permit additional training time and visits to outlying islands. Weekly or biweekly teleconferences are scheduled so that they do not disrupt local clinical requirements. A local programme coordinator has been hired to provide physician support and to ensure patients are scheduled at times convenient for them, with appropriate follow-up. Nutritional interventions and educational pamphlets have been developed and distributed to address misconceptions about hepatitis and lessen the stigma associated with it. The provision of laboratory testing (HDV antibody and both HBV and HDV viral load) by VIDRL is a temporary fix, so there is a focus on laboratory capacity-building, training and skills transfer. Input from local medical personnel has guided the development of data management tools, such as clinical spreadsheets, that greatly improve the efficiency of patient encounters, are much simpler to learn, and minimize the time required to maintain and analyse data.

Co-infection with hepatitis B and D remains a significant problem since there is not yet an effective treatment. Currently, interferon is the only recommended therapy, but it is not practical to use in this setting. There are promising new drugs on the horizon, but they are still undergoing clinical trials. (*11*) Prevention is by far the best strategy to prevent the co-infection with hepatitis B and D. To this end, birth-dose hepatitis B immunization is being prioritized, and a new national plan to treat expectant mothers who have CHB and high viral loads has been approved. The latter strategy has been found to be effective in the prevention of mother-to-child transmission of hepatitis B in other settings. (*12*) Given the resource limitations of the country, funding to support this programme to reduce the rate of vertical transmission was sought and has been secured.

## Discussion

Hepatitis B causes considerable morbidity, mortality and economic loss in the Pacific islands. Although there is not a cure, its effects can be ameliorated by the implementation of proven prevention strategies and national treatment programmes using effective antiviral therapy for those already infected. In the short time since implementation of the treatment programme in Kiribati, patients who remain adherent to therapy have reported an overall improvement in well-being. Progress has been made but has been hampered by the problems described above, each of which is being actively addressed. Remaining cognizant of the local needs, we are optimistic for an acceleration in patient recruitment and treatment.

Kiribati has embraced the need to finally address CHB. There is considerable appetite to establish similar programmes in other Pacific island nations. Working with VIDRL and WHO, HBF is currently providing medications, laboratory support and training to jump-start pilot treatment programmes in other Pacific island countries such as Fiji, Tonga and Vanuatu. Discussions are also under way to implement treatment programmes to interrupt maternal-to-child transmission in these countries.

Lack of infrastructure and training, co-infections, geographic considerations, limited public knowledge and social norms create problems that are likely shared in other low- and middle-income settings, particularly small islands. Nevertheless, there is no one-size-fits-all solution for every country. Treatment programme development has required patience, close engagement with local partners, cultural sensitivities, a significant time investment and attention to the needs of the population served. Programme administrators need to “get their hands dirty” and observe first-hand the work being done on the front lines. With innovative strategies to deliver services, testing and effective treatment of CHB can be provided to LMICs such as Kiribati.
